# Experimental study on fractal dimension of energy dissipation and crack growth in saturated tuff at different strain rates

**DOI:** 10.1038/s41598-024-64273-4

**Published:** 2024-06-10

**Authors:** Hao Wang, Qi Zong, Ziyi Lu, Haibo Wang

**Affiliations:** 1 School of Civil Engineering and Architecture, Anhui University of Science and Technology, Huainan, 232001 Anhui China; 2 China Construction Sixth Bureau Eighth Construction Co., LTD, Hefei, 230041 Anhui China

**Keywords:** Strain rate, Tuff, Full of water, Crushing energy density, Crack propagation, Fractal dimension, Civil engineering, Hydrology

## Abstract

In order to investigate the effects of strain rate and water saturation on the energy dissipation and crack growth of tuff, uniaxial compression tests were carried out on dry and water saturated tuff with different strain rates using an electro-hydraulic servo press and a 50 mm diameter split Hopkinson pressure rod (SHPB) device. High-speed camera and Image J image analysis software were used to obtain the crack growth process of the specimen under impact load, and fractal dimension was introduced to quantitatively study the crack growth degree. The results show that more than 90% of the energy is stored in the specimen as elastic energy when it reaches the peak stress under static load. The average total energy of water-saturated specimens is 67.55% of that of dry specimens. The average energy dissipation density of water-saturated specimens under 0.3 MPa, 0.4 MPa and 0.5 MPa air pressure is 0.79, 0.91 and 0.92 times of that of dry specimens, respectively. Water-saturated specimens will deteriorate and thus reduce their energy storage and energy absorption effects. The reflected energy, transmitted energy, absorbed energy and incident energy are linear, logarithmic and linear functions, respectively, and the energy absorptivity and specific energy absorptivity of water-saturated specimens are lower than those of dry specimens. Due to the existence of “stefan” effect, the increase of energy dissipation density of water-saturated specimen at high strain rate is greater than that of dry specimen. The mean fractal dimension of water-saturated specimens under 0.3 MPa, 0.4 MPa and 0.5 MPa is 1.09, 1.05 and 1.16 times that of dry specimens. At the same strain rate, the number and width of cracks in water-saturated specimens are larger than that in dry specimens. Water-saturated behavior reduces the energy absorption capacity of tuff, increases the fractal dimension of crack growth, and significantly reduces the resistance of water-saturated rock to external loads.

## Introduction

The safety problem of mine is the most concerned core factor in the process of resource exploitation, and also the key factor that restrains the development of engineering economy^1,2^. Since the new century, along with the improvement of industrial science and technology, the mining speed and mining technology of mine resources have been rapidly improved^3,4^. Meanwhile, geological disasters induced in the mining process are increasing day by day, which also puts forward higher requirements for corresponding protective measures^5,6^. In addition to the stability of rock mass under conventional static load, the reliability of rock mass engineering under dynamic load such as flood, rockburst, earthquake and blasting should be fully considered^7–9^. As the most common fluid in nature, water affects many rock masses. In nature, there are a large number of defects such as primary joints and cracks in the rock mass itself. Under the action of water pressure, the cracks will be filled with free water, thus changing the mechanical properties of the rock mass itself^10–13^. Under the action of load, cracks in the weak layer within the rock mass develop, expand and connect first, and energy is dissipated during this process. However, the changes of rock mass mechanical properties and the process of rock mass breakage under load are extremely complex, and there will be a large error in estimating and measuring the difficulty of rock mass breakage under load only by using compressive strength. While energy conversion is the essential feature of physical reaction of material^14^. Through the analysis of the energy dissipation process of rock mass, the whole process of rock mass damage and failure can be reflected, which is of great value for the analysis of rock mass mechanical properties and fracture mechanism under load.

In recent years, many experts and scholars have studied the energy dissipation law of different rock mass under dynamic load, thus reflecting the deformation and failure process of rock mass. Jia et al.^15^ used SHPB device to study the mechanical properties and energy dissipation law of granite under single and repeated impact loads, and analyzed the degree of fragmentation of rock mass under impact loads from the perspective of energy. Li et al.^16^ studied the energy evolution law of heterogeneous coal samples at different loading rates, and derived the energy dissipation damage constitutive model of specimens. Wang et al.^17^ studied the energy dissipation law and fracture fractal characteristics of coal samples with different length-diameter ratios under impact load, and analyzed the effect of length-diameter ratio on energy dissipation of specimens and the relationship between length-diameter ratio, energy dissipation density and fracture fractal characteristics. Zhang et al.^18^ studied the energy dissipation and fracture patterns of mudstone under impact load and different stress states, and analyzed the laws between impact load, axial pressure, confining pressure and the characteristic values of each energy and absorbed energy. Fang et al.^19^ studied the law of energy dissipation and damage evolution of limestone under impact load, and discussed the relationship between damage variables of specimens and energy dissipation density based on Weibull damage theory. Many research results have analyzed the damage and failure processes of different rock masses from the perspective of energy, which will better reveal the damage and failure processes of rock masses under external loads from the essential aspect, and have great significance for studying the changes of mechanical properties of rock masses under dynamic loads.

Due to the influence of the environment where the project is located, many mining projects are in a state of saturated water for a long time, such as heavy precipitation areas, hydropower stations and other engineering areas. The existence of water in rock mass will affect the properties of tuff itself^20–23^, thus greatly affecting the mechanical properties and fracture characteristics of rock mass under dynamic load. For the influence law of saturation action on energy dissipation of rock mass under dynamic load, Jin et al.^24^ used SHPB device to study the fracture characteristics and energy dissipation law of red sandstone with different water content under impact load. The results showed that the energy dissipation rate of the specimen first increased with water content and then decreased, and the degree of breakage increased with the increase of water content. Song et al.^25^ used the true triaxial SHPB loading system to study the energy dissipation characteristics of natural and water-saturated coal samples, and the research results showed that water-saturated action would reduce the energy storage capacity of coal and its ability to resist external loads. Liu et al.^26^ studied the energy dissipation law of water-saturated SLATE under impact load and found that water-saturated action would enhance the dissipated energy density of SLATE. In the study on the influence of water saturation on the crack growth law of rock mass under dynamic loading, Wang et al.^27^ studied the crack growth law of red sandstone under static and dynamic loading under water saturation, and the results showed that free water would hinder the crack growth under impact load, thus inhibiting the crack growth. Zhang et al.^28^ used the digital image correlation method to study the influence of the precursor abnormal characteristics of fractured sandstone under the action of water saturation. The results showed that the water saturation would aggravate the non-uniformity of rock mass and make the crack initiation, propagation and penetration process of specimen faster than that of natural specimen. Saturation action can promote crack growth under load, but because the process of crack growth in rock mass is relatively discrete, no unified theory has been formed on the law of crack growth. Some scholars have introduced fractal theory into rock micro-fracture and macro-crack dynamic growth to quantitatively study the complexity of rock crack growth under loads, which also provides a new direction for the analysis of rock crack growth laws under different loads^29–31^.

At present, there are few researches on the mechanical properties of tuff samples under water-saturated action, and most of the existing achievements are the macroscopic mechanics analysis of water-saturated rock mass, and few involve the law of crack propagation and microstructure change during the failure process of rock mass. This research is based on the mining project of Dahuangshan building stone (tuff) mine in Dinghai District, Zhoushan City. The project site is located at the seaside and has abundant rainfall all the year round. On one side of the mountain, there are more than 10 high and steep slopes left by the previous mining. In the current blasting mining process, the rock slope needs to bear a variety of static loads and dynamic loads (its own mass, tidal shaking, seismic action, various mining tools, blasting loads). In order to explore the effects of loading rate and water saturation on energy dissipation and crack propagation of tuff in engineering site, the electro-hydraulic servo press and SHPB test device were used to carry out uniaxial compression tests at different strain rates on dry and water saturated specimens. The crack growth process of specimens under impact load was obtained by combining high-speed video system and Image J image analysis software. The fractal dimension of crack growth under the box dimension is introduced to quantitatively describe the crack of the specimen. The effect of water saturation and strain rate on energy dissipation and crack propagation process of tuff in engineering site is analyzed. The research results can provide an experimental basis for formulating reasonable engineering measures and ensuring the safety and stability of engineering rock mass in tuff mining projects.

## Test

### Preparation of specimen

**Figure 1:**
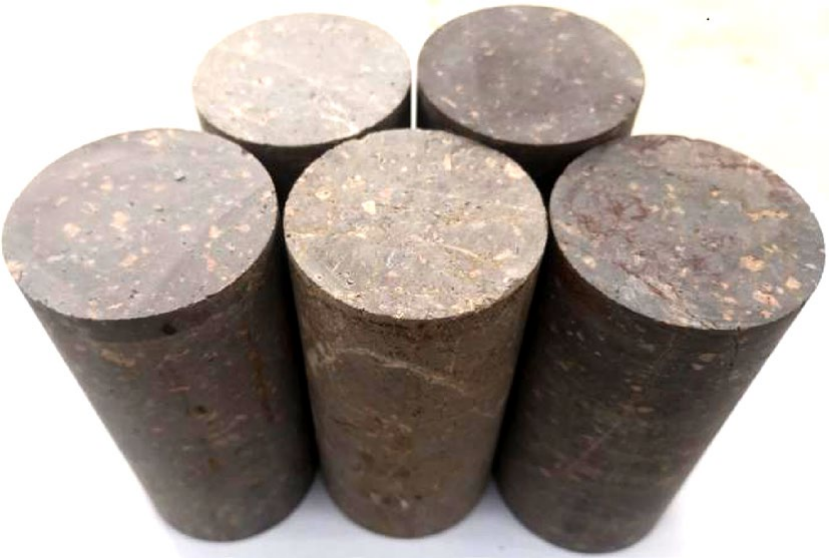
Partial tuff sample.

Tuff specimens were selected from Zhoushan engineering site in Zhejiang Province. In order to ensure the similar physical properties of the selected specimens, the specimens were taken from the same uniform and complete sheet. According to the International Society of Rock Mechanics (ISRM) standard, the on-site rock mass was processed into standard cylinder specimens with diameter of 50 mm and length of 100 mm and diameter of 50 mm and length of 25 mm. Some of the specimens were shown in Fig. 1, and the roughness of their end surface was within 0.02 mm ^32^. Ultrasonic detector was used to select specimens with relatively concentrated P-wave velocity for testing to reduce the error caused by the dispersion of rock samples to the test results^33^. Some of the physical and mechanical properties are shown in Table 1.

**Table 1:** Static physical and mechanical properties of tuff.

Density/(g·cm^−3^)	Longitudinal wave velocity/(m·s^−1^)	Mean porosity/%	Compressive strength/MPa
2.67	4425	2.23	52.92

After the specimen is processed, the specimen is saturated with water according to SL/ T264-2020 "Rock Testing Regulations for Water Conservancy and Hydropower Engineering". First, the processed specimen is placed in a drying oven for 48 h for drying treatment, and then the dried specimen is prepared after natural cooling to room temperature^23^. Vacuum water saturation device was used to treat some dry specimens with vacuum water saturation. When saturated, the water surface in the container is higher than the upper surface of the specimen, the degree of the vacuum pressure dial is 0.1 MPa, and the pumping time of the saturated sample is 6 h. After the end of air pumping, the specimen was placed in water for 30-day curing, during which the water surface was ensured to be 2 cm higher than the rock surface^34^.

### Test loading device and principle

The static loading device uses an electro-hydraulic servo press and adopts displacement controlled loading. The strain rate is calculated as shown in Eq. (1)^35^.

1 $$\dot{\varepsilon } = \frac{v}{L}$$

where: $\dot{\varepsilon }$ is strain rate, s^-1^; *v* is the loading rate, m/s; *L* is the height of the specimen, m.

Ignoring the energy exchange between tuff and the outside during static loading, and considering only the axial stress–strain of rock mass, the total input strain energy, elastic strain energy and dissipated energy of specimens in the stress space can be expressed as^36^ :

2 $$U = U^{e} + U^{d}$$

3 $$U = \int\limits_{0}^{\varepsilon } \sigma {\text{d}}\varepsilon$$

4 $$U^{e} = \frac{1}{2}\sigma \varepsilon^{e} = \frac{1}{{2E_{{\text{u}}} }}\sigma^{2}$$

where: *U* is the total work done by external force, J; *U*^e^ is the elastic deformation energy stored in the specimen, J; *U*^d^ is used to damage specimens and generate plastic deformation energy in the process of loading, J; *σ* is the stress of the specimen, MPa; *ε* is the strain of the specimen, which is dimensionless. *E*_U_ is the elastic modulus of the specimen, GPa.

**Figure 2:**
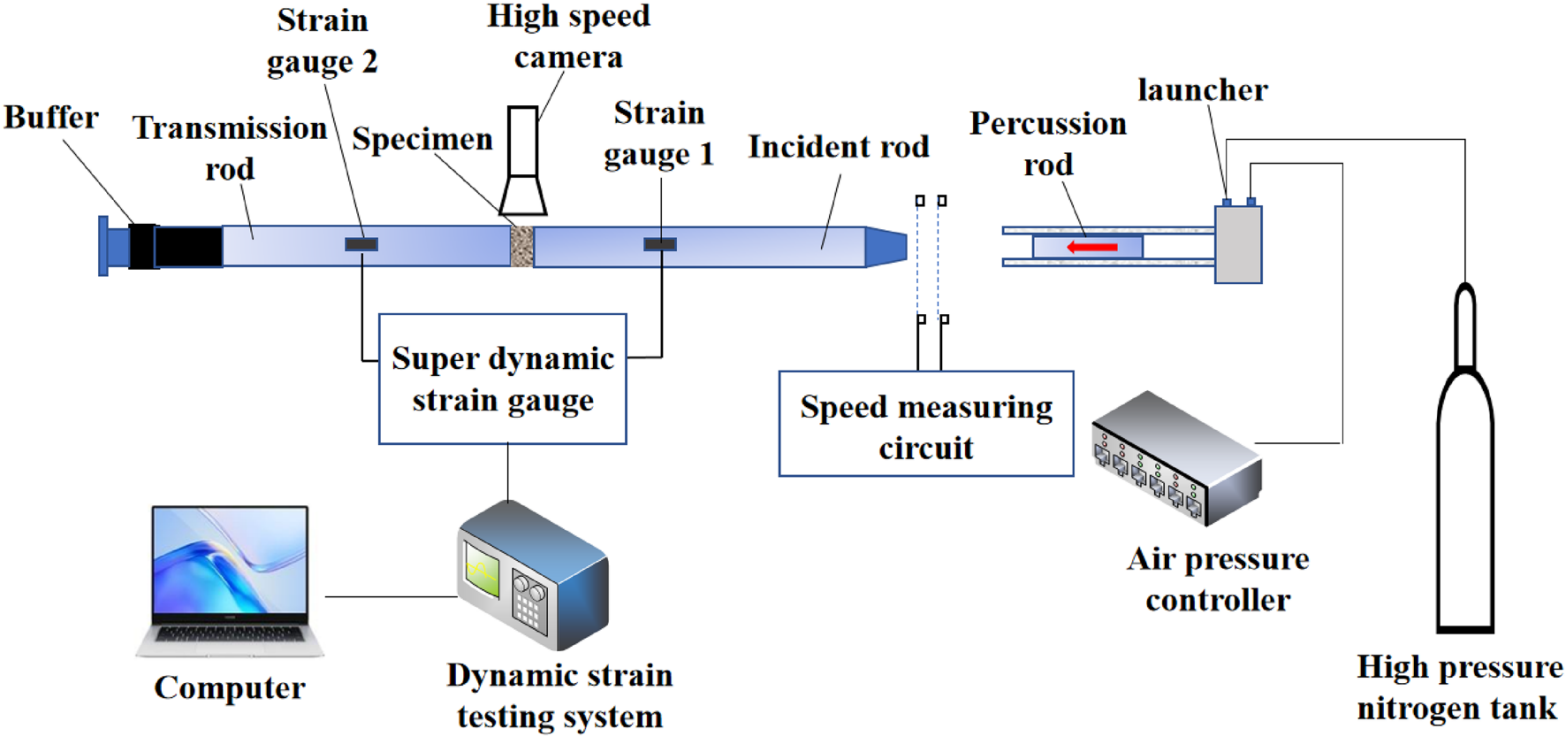
SHPB test device diagram.

The dynamic loading device uses the separated SHPB test system with a diameter of 50 mm. The structure of the device is shown in Fig. 2. Studies show that when tapered punch is used for loading, the loading waveform has no oscillation or the degree of oscillation is small, thus reducing the influence of the loading waveform oscillation on the test results^37^. The device system includes impact bullet, incident rod, transmission rod, damping device and data acquisition system. The length of the bullet, the incident rod and the transmission rod are 0.6 m, 2.4 m and 1.2 m, respectively. They are all made of alloy steel with a density of 7850 kg/m^3^, an elastic modulus of 210 GPa and a P-wave velocity of 5190 m/s. A BX120-3AA resistance strain gauge with a value of 120 ± 0.2Ω and a sensitivity factor of 2.08 ± 1% is pasted on both the incident rod and the transmission rod. The signal acquisition devices are ultra-dynamic strain gauges and oscilloscopes.

**Figure 3:**
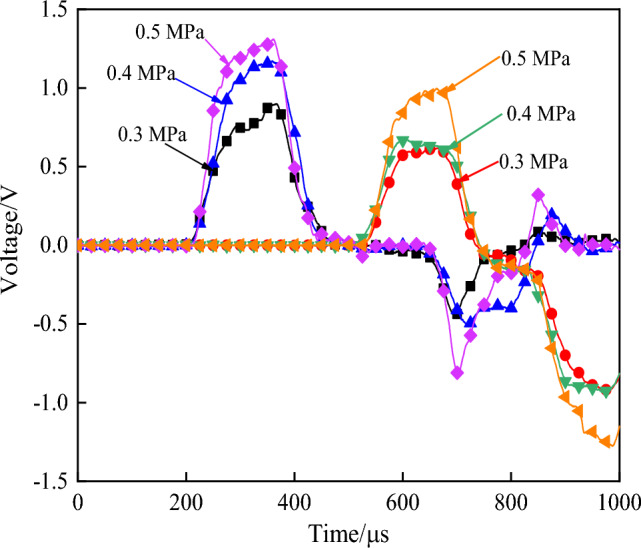
Measured waveform.

**Figure 4:**
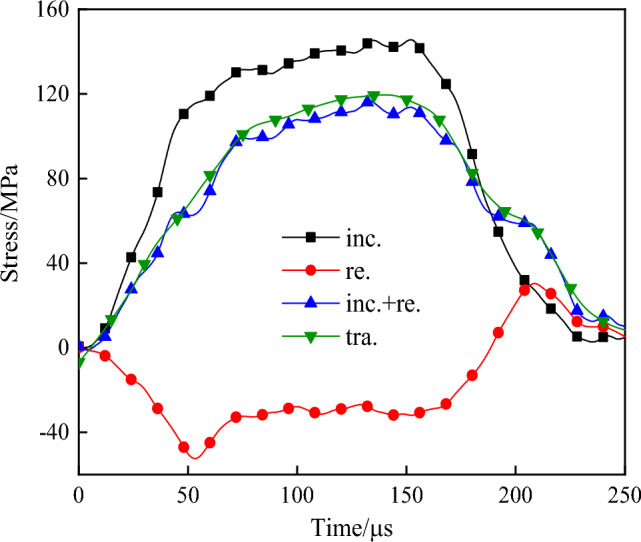
Stress equilibrium curve.

Under the action of high pressure gas, the bullet hits the incident bar at a certain initial velocity to form an incident wave. The incident wave propagates forward along the incident bar, but due to the difference in wave impedance between the bar and the specimen, the wave will be reflected and transmitted on the contact surface of the two. The reflected wave signal is received by the strain gauge on the incident bar, and the transmitted wave is received by the strain gauge on the end of the transmission bar. The original waveform obtained under different impact pressure is shown in Fig. 3. Due to attenuation of the stress wave along the transmission direction, there would be a certain stress difference when the wave reached both ends of the specimen for the first time. However, with continuous reflection of the wave, the stress difference between the two ends of the specimen would decrease continuously until it basically disappeared, and the stress at both ends would reach a state of equilibrium^37^. In order to ensure the effectiveness of impact test data, stress balance detection should be carried out on each impact data, and the stress balance curve is shown in Fig. 4.

According to the one-dimensional elastic wave theory, the incident energy *W*_i_, reflected energy *W*_r_, transmitted energy *W*_t_ and dissipated energy *W*_s_ of the specimen under impact load can be obtained, and the relevant calculation formula is as follows ^38^:

5 $$\left\{ \begin{gathered} W_{i} (t) = AEC_{0} \int_{0}^{t} {\varepsilon_{i}^{2} (t)dt} \hfill \\ W_{r} (t) = AEC_{0} \int_{0}^{t} {\varepsilon_{r}^{2} (t)dt} \hfill \\ W_{t} (t) = AEC_{0} \int_{0}^{t} {\varepsilon_{t}^{2} (t)dt} \hfill \\ \end{gathered} \right.$$

6 $$W_{s} (t) = W_{i} (t) - W_{r} (t) - W_{t} (t)$$

where: *W*_i_、*W*_r_ 、*W*_t_、*W*_s_ is the incident energy, reflected energy, transmitted energy and dissipated energy at time t, J; $\varepsilon_{{\text{i}}} (t)$、$\varepsilon_{{\text{r}}} (t)$、$\varepsilon_{{\text{t}}} (t)$ are incident strain, reflection strain and transmission strain at time t, and they are dimensionless. $A,E$ is the cross-sectional area and elastic modulus of the pressure bar, respectively, m^2^ and MPa; *C*_0_ is the P-wave velocity of the pressure bar, m/s.

According to the dissipated energy of the sample, the energy dissipation rate $\omega_{d}$ and the crushing energy dissipation density $\varepsilon_{d}$ of the sample are defined as follows:

7 $$\omega_{d} = \frac{{W_{s} (t)}}{{W_{i} (t)}}$$

8 $$\varepsilon_{d} = \frac{{W_{s} (t)}}{V}$$

where: $\varepsilon_{d}$ is crushing energy dissipation density, J/cm^3^; *V* is the specimen volume, cm^3^.

### Process of test

After the saturation test, the static uniaxial compression test of dry and saturated tuff specimens was carried out by electro-hydraulic servo press, and the loading rate was controlled at 0.01 mm/s. Dynamic uniaxial compression test uses SHPB test device system. In order to ensure that the specimen can be broken under impact load and better compare the influence of saturation on the specimen's fracture morphology, test punching is carried out on the specimen before the test. Finally, three kinds of impact pressure (0.3 MPa, 0.4 MPa and 0.5 MPa) were selected to obtain different strain rates, and the crack growth process of dry and saturated tuff specimens under impact loads was obtained by combining with high-speed camera technology. The image acquisition time interval of high-speed camera is 20 µs. Image J image analysis software was used to extract the complete path of specimen crack growth process under impact load, and fractal dimension of crack growth was introduced to conduct quantitative analysis of the complexity of specimen crack growth^39^. During the test, select three test pieces for each group, and finally select the two data closest to the average value for analysis.

## Results and discussion

### Energy curves of specimens under static and dynamic loads

**Figure 5:**
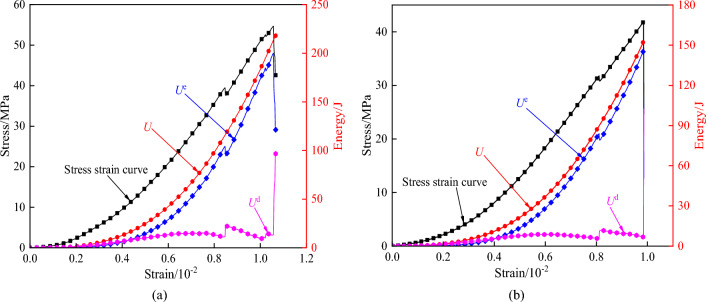
Energy change curve of rock mass under static load. (**a**) Energy change curve of dry specimen, (**b**) Energy change curve of saturated specimen.

**Figure 6:**
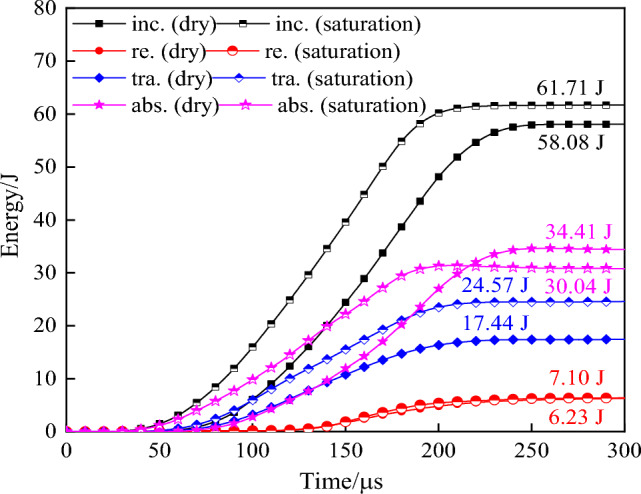
Time history curve of rock mass energy under 0.3 MPa air pressure.

Uniaxial compression tests with different strain rates were carried out on dry and saturated tuff samples. The energy changes of specimens under static load were shown in Table 2. The energy variation curve under load is shown in Fig. 5. The energy changes of specimens under impact load are shown in Table 3. The energy time history curve of specimens under 0.3 MPa air pressure is shown in Fig. 6.

**Table 2:** Each energy index at the peak point of the specimen under static load.

Specimen number	State	Mean strain rate/s^-1^	Peak stress/MPa	Total energy/J	Elastic deformation energy/J		Plastic deformation energy/J	
					Numerical	Proportion/%	Numerical	Proportion/%
JY1-1	dry	10^–4^	54.66	218.27	199.94	91.60	18.33	8.40
JY1-2	dry	10^–4^	51.18	226.78	206.41	91.02	20.37	8.98
JY2-1	saturation	10^–4^	41.75	151.96	145.14	95.51	6.82	4.49
JY2-2	saturation	10^–4^	39.55	148.68	140.59	94.56	8.09	5.44

(JY1-1: JY stands for static compression test piece, 1–1 stands for test piece No. 1–1).

**Table 3:** Uniaxial compression test results under impact load.

Specimen number	State	Average strain rate/s^-1^	Peak stress/MPa	Incident energy/J	Reflection energy/J	Transmissive energy/J	Absorbed energy/J	Energy dissipation rate/%	Crushing energy consumption density/J.cm^-3^
DY1-1	dry	101.3	61.93	58.08	6.23	17.44	34.41	59.25	0.702
DY1-2	dry	104.2	65.24	63.76	10.33	17.35	36.08	56.59	0.735
DY1-4	dry	128.5	77.99	88.74	17.48	23.41	47.85	53.92	0.975
DY1-6	dry	126.5	75.69	88.68	19.86	24.88	43.94	49.58	0.794
DY1-8	dry	156.3	101.35	108.48	25.16	32.33	50.99	47.01	1.039
DY1-9	dry	159.5	106.02	107.07	21.51	35.15	50.41	47.08	1.027
DY2-1	saturation	105.2	55.38	57.98	11.67	20.51	25.80	44.50	0.526
DY2-2	saturation	102.8	58.36	61.71	7.10	24.57	30.04	48.68	0.612
DY2-4	saturation	132.3	76.82	79.80	13.17	26.92	39.71	49.76	0.809
DY2-5	saturation	130.1	80.32	80.76	12.12	28.18	39.46	48.86	0.804
DY2-7	saturation	155.7	110.46	101.99	20.36	33.61	48.02	47.08	0.979
DY2-9	saturation	158.6	113.56	101.04	22.25	37.34	41.45	41.02	0.923

(DY2-1: DY represents dynamic compression test piece, 2–1 represents test piece No. 2–1).

Combined with Table 2 and Fig. 5, it can be seen that under static load, all the energy of the specimen increases with the increase of the loading time, and more than 90% of the energy of the specimen at the peak stress is stored in the specimen in the form of elastic energy. When the total energy of the specimen is no longer increased after it reaches the peak stress, the elastic deformation energy decreases rapidly while the plastic deformation energy increases. At this time, the specimen has sudden brittle failure, and the elastic deformation energy stored in the specimen is rapidly transformed into the plastic deformation energy. By comparing the energy changes of dry and water-saturated specimens, it can be seen that the mean value of the total energy of water-saturated specimens under static load is 67.55% of that of dry specimens, and the proportion of elastic deformation energy of water-saturated specimens is higher than that of dry specimens. This is because water saturation will cause deterioration of tuff, thus reducing the energy storage effect of test pieces. Deterioration mainly includes three aspects^40,41^. (1) Under static load, free water in rock crack will form water pressure at the crack tip to accelerate the further propagation of crack, which makes the specimen more prone to failure. (2) The water–rock erosion will cause the dissolution of part of the solute inside the specimen and increase its porosity. (3) Under water erosion, the bonding effect between mineral particles of the specimen is weakened, and the friction force is reduced, thus reducing its ability to resist external loads. The deterioration reduces the energy storage effect of the specimen.

It can be seen from Fig. 6 that the internal energy of the specimen under impact load increases with the increase of load acting time. The action of each energy in the specimen can be roughly divided into three stages according to the time. (1) The specimen within 0 ~ 75 µs is in the elastic deformation stage. During the compression process, the internal cracks of the specimen are compressed, and the energy absorbed inside is stored in the form of elastic energy, with little variation amplitude of each energy. (2) Each energy rises in a linear manner within 75 ~ 200 µs. Due to the difference in wave impedance between the rock and the pressure rod, part of the energy on the contact surface of the two is reflected back to the pressure rod, part of the energy passes through the rock to the transmission rod, and the rest of the energy is absorbed by the specimen for its internal plastic deformation. (3) After 200 µs, each energy increases slightly and tends to a fixed value, and the action time of each energy on the specimen ends, and the specimen becomes stable again. Under 0.3 MPa impact pressure, the peak time of each energy of the saturated specimen is shorter than that of the dry specimen, and the transmission energy and absorption energy of the saturated specimen are 1.41 times and 0.87 times of that of the dry specimen. Combined with the propagation law of one-dimensional stress wave, it can be seen that when the stress wave is transmitted to the contact surface of the bar and the specimen, the permeability reflection coefficient of the specimen is related to the wave impedance of both. The smaller the ratio between the wave impedance of the specimen and the wave impedance of the rod, the higher the transmission coefficient. Because the rock mass density and p-wave velocity increase under the action of water saturation, the wave transmission velocity and transmission coefficient increase, and the time for each energy to reach the peak decreases and the transmission energy increases^42,43^. Due to the erosion and degradation of water, the energy absorption effect of saturated specimen is lower than that of dry specimen.

### The relationship between energies under impact loads

**Figure 7:**
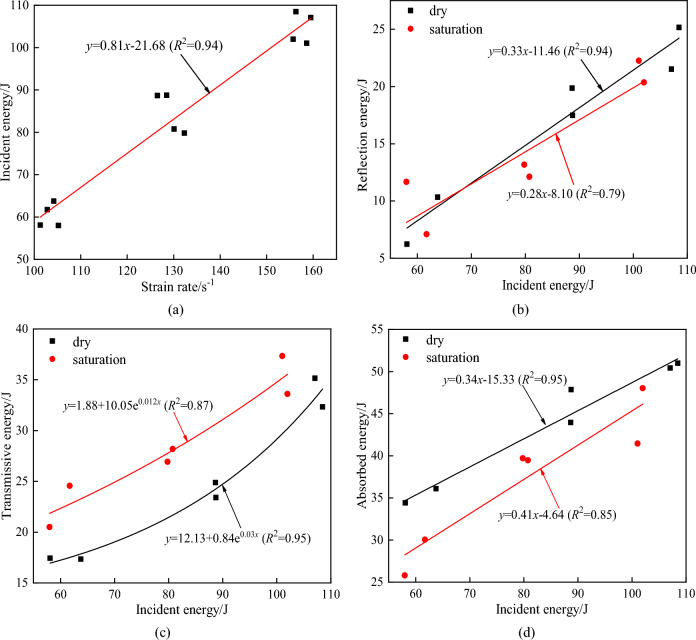
The relationship between the energies. (**a**) Relation between strain rate and incident energy, (**b**) Relation between reflected energy and incident energy, (**c**) Relation between transmitted and incident energy, (**d**) Relation between absorbed and incident energy.

In order to further explore the relationship between various energies under impact load, the changes of various energies under different strain rates are fitted. The relationship between various energies is shown in Fig. 7.

In the test, the bullet hits the incident bar at a certain speed under the action of high-pressure nitrogen. The energy loss in the impact process is ignored, and the impact kinetic energy is converted into the incident energy. It can be seen from Fig. 7(a) that there is a good linear relationship between the incident energy of the specimen and the strain rate, which also proves the validity of the test data under impact^44^. Due to the inconsistency between the impedance of the specimen and the pressure rod wave, some stress waves will be reflected on the contact surface of the two. There is a good linear positive correlation between the reflected energy and the incident energy. The reflected energy of saturated rock is slightly lower than that of dry rock. Transmission energy increases with the increase of incident energy, and its incremental relative value increases accordingly. There is a good logarithmic function relationship between the two. The transmission energy of the saturated rock is obviously higher than that of the dry specimen, and the propagation speed of the longitudinal wave velocity of the saturated specimen is higher than that of the dry specimen. The difference between the wave impedance and the pressure rod is smaller than that of the dry specimen, which increases the transmission of the transmitted wave to a certain extent. There is a good linear positive correlation between the absorption energy of the specimen and the incident energy. The absorption energy of the dry specimen is higher than that of the saturated specimen, and the saturation effect reduces the energy absorption effect of the specimen.

**Figure 8:**
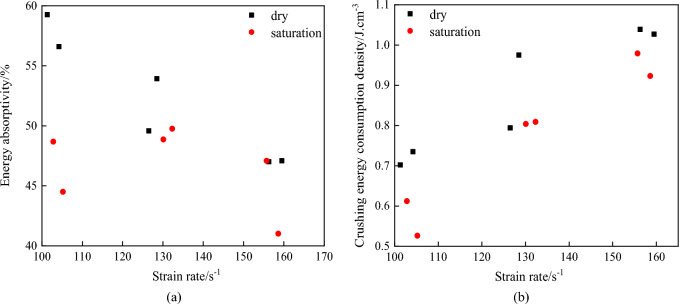
Relation between strain rate and energy absorption effect. (**a**) Relation between strain rate and energy absorption, (**b**) Relation between strain rate and energy absorption.

In order to further explore the energy absorption effect of dry and water-saturated rock specimens under different strain rates, energy dissipation rate and crushing energy dissipation density were introduced. The relationship between strain rate, energy dissipation rate and crushing energy dissipation density is shown in Fig. 8.

It can be seen from Fig. 8a that the energy absorption rate of dry specimen under different strain rates is higher than that of saturated specimen. With the increase of strain rate, the energy absorption rate of the specimen decreases. It can be seen from Fig. 8b that with the increase of strain rate, the breakage energy dissipation density of specimens increases, and the breakage energy dissipation density of dry specimens is higher than that of saturated specimens. When the strain rate is about 102 s^−1^, the average energy dissipation density of saturated rock sample is 79.2% of that of dry rock sample. The average energy consumption density of saturated rock sample at 155 s^-1^ is 92.1% of that of dry rock sample. The increase of energy consumption density of saturated rock sample is higher than that of dry rock sample, which indicates that the increase of strain rate enhances the energy consumption effect of saturated rock sample. Saturated rock mass deteriorates and its energy absorption rate and energy dissipation density are lower than those of dry specimens. When the strain rate increases, on the one hand, the water film adsorbed on the pore wall and the surface of rock and mineral particles will generate cohesive force which hinders crack growth during the rapid crack expansion process. On the other hand, due to the "stefan" effect and the existence of free water under high strain rate, the specimen will produce a reaction force to restrain the rock mass from separating up and down^45,46^. The existence of the two forces will consume part of the energy, and the greater the strain rate, the greater the force formed, the increase of the energy consumption density of the saturated specimen will increase.

### Crack propagation process of specimen under impact load

**Figure 9:**
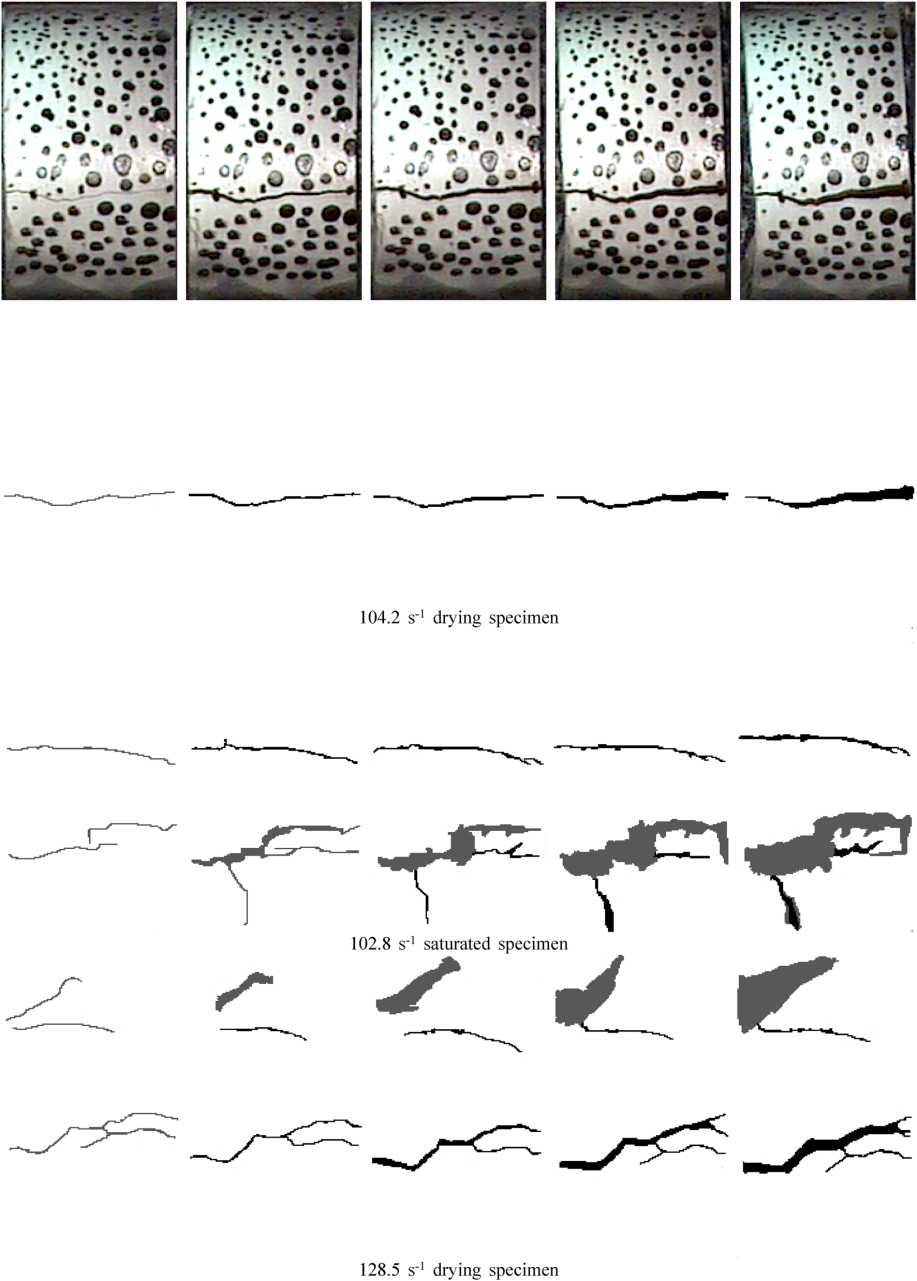

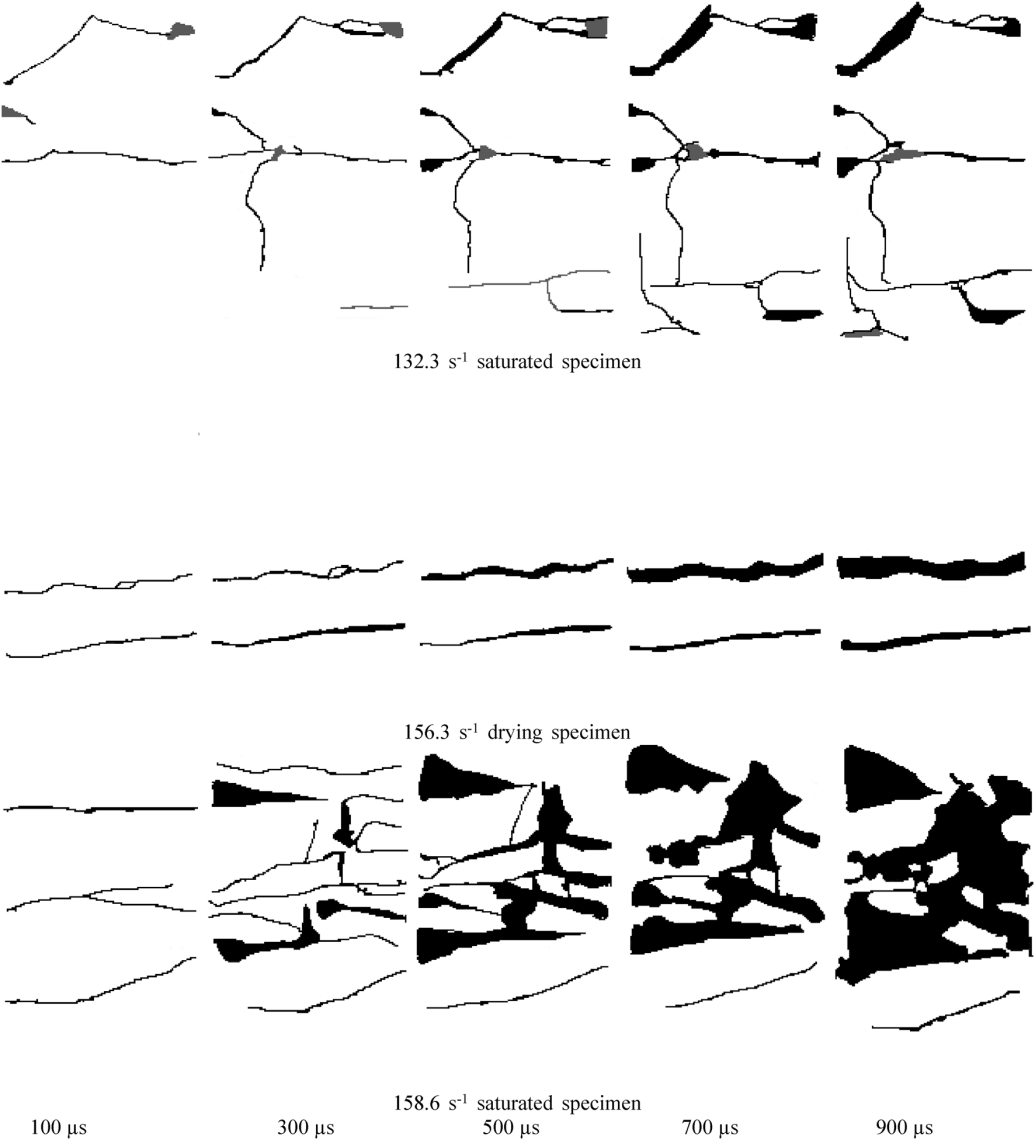
Crack growth process of specimen under impact load.

The crack growth and failure processes of dry and water-saturated specimens under different impact pressures were obtained by high-speed imaging technology, and the crack growth processes were extracted by Image J image analysis software. The process of crack growth with time under different impact pressures is shown in Fig. 9.

It can be seen from Fig. 9 that the specimen produces plastic damage deformation under the external impact load, which is manifested in the propagation of cracks (black) and spalling of surface rock mass (gray). With the increase of the time of impact load, the crack propagation occurs, and the width and number of cracks increase. Under the action of external load, the specimen absorbs part of the energy for its own damage and deformation, which is manifested in the microscopic form as the separation of the cohesive particles inside the specimen, and in the macroscopic form as the expansion of cracks and the fracture of the specimen. With the increase of strain rate, the crack propagation degree of dry and water-saturated specimens increases significantly, which is manifested as the increase of crack width and number. Combined with Figs. 7 and 8, it can be seen that the increase of strain rate increases the absorption energy of the specimen, increases the crushing energy dissipation density, and increases the plastic damage and breakage degree of the specimen itself.

Under the same strain rate, the fracture forms of dry and saturated tuff specimens are significantly different. Under the same strain rate, the fracture degree of dry specimen is obviously lower than that of saturated specimen. At 104.2 s^−1^ strain rate, only one main crack was generated in the dry specimen. With the increase of loading time, the crack width increased continuously, and the specimen finally broke along this main crack. At 102.8 s^-1^ strain rate, the number of cracks in the saturated specimen is significantly more than that in the dry specimen, and the surface rock mass of the specimen is spalling. When the strain rate is 130 s^−1^, the number of cracks in the saturated specimen is significantly more than that in the dry specimen. When the strain rate is 156 s^−1^, the dry specimen is broken along the two main cracks, while the saturated specimen is "burst" under this strain rate, and the crack of the specimen rapidly expands and the debris splashes. The results show that the degree of breakage of the saturated specimen is significantly higher than that of the dry specimen under the same strain rate. Combined with Fig. 8, it can be seen that the crushing energy dissipation density of water-saturated specimens is lower than that of dry specimens, indicating that the energy-absorbing effect and the resistance to external load deformation of water-saturated specimens are significantly lower than that of dry specimens. The erosion softening effect of water on rock mass under saturation greatly reduces the deformation ability of rock mass under external load, which reduces the bonding effect among the cohesive particles inside the rock mass and makes it more prone to plastic deformation under external load.

### Fractal dimension of crack under the principle of box dimension

**Figure 10:**
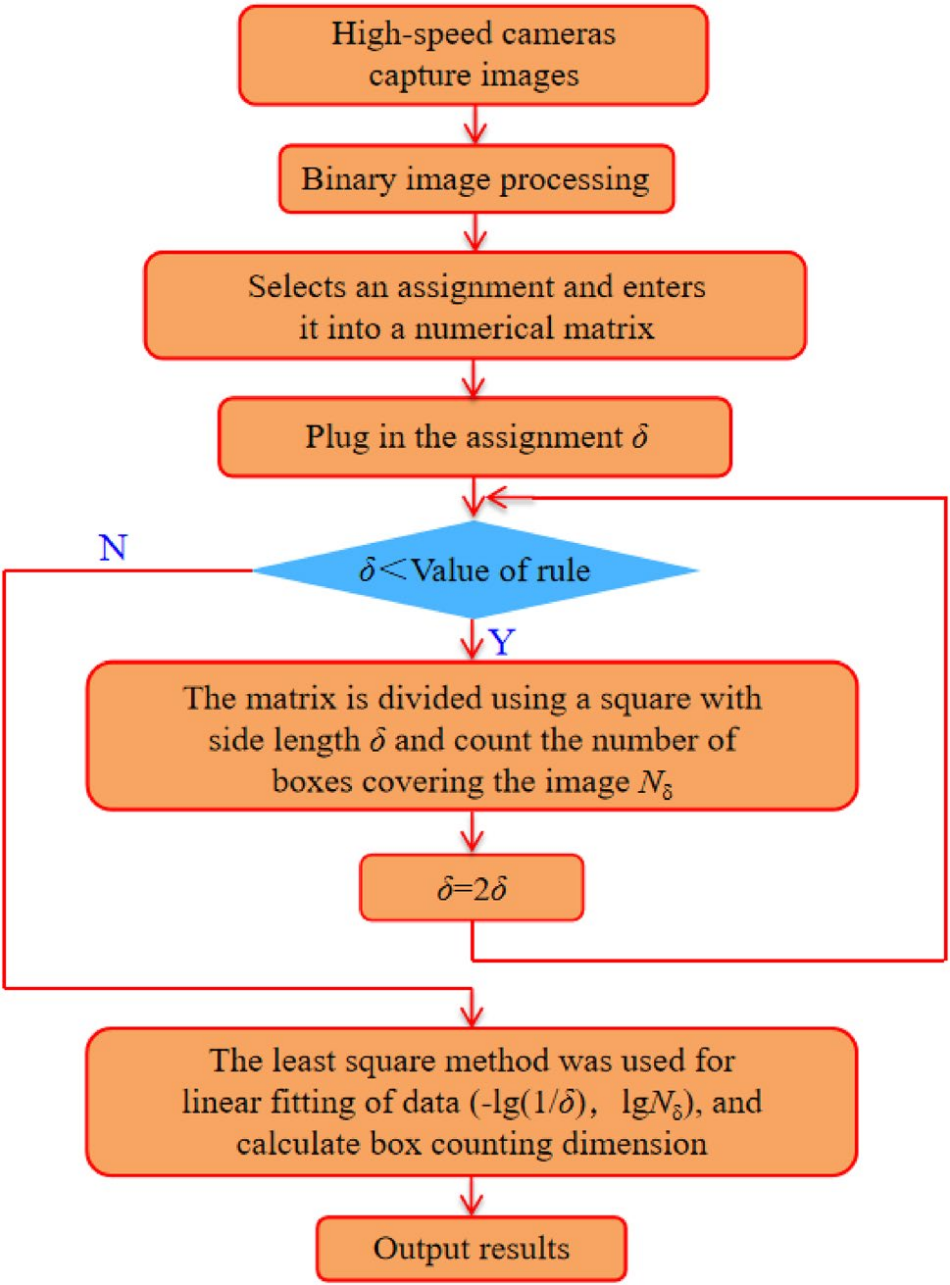
Box counting dimension calculation flow chart.

In order to further quantitatively analyze the crack growth degree of dry and saturated specimens, fractal dimension *D* of crack growth was introduced. Using MATLAB program software to calculate the box dimension, the image of crack growth process was binarized, and the obtained binarized image was divided by the equivalent grid of side length *δ*. Based on the principle of box dimension (Eq. (9)), the fractal dimension of dry and saturated tuff samples under different impact pressures can be calculated. The program flow is shown in Fig. 10, where *F* is any non-empty bounded subset, *N*_δ_(*F*) is the number of boxes with side length *δ* intersecting *F*, and the box dimension of set F can be obtained by dim_B_*F*^47,48^.

9 $$\dim_{B} F = \mathop {\lim }\limits_{\delta \to 0} \frac{{\lg N_{\delta } (F)}}{ - \lg (1/\delta )}$$

**Figure 11:**
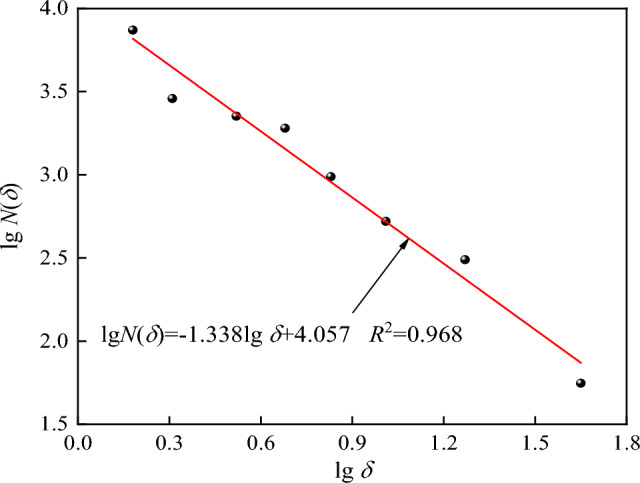
Calculation method of fractal dimension in crack propagation process of specimen.

**Figure 12:**
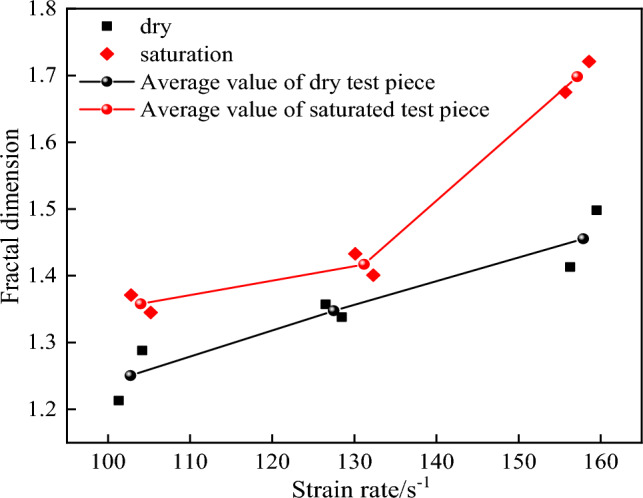
Fractal dimension of dry and water-saturated specimens under different shock pressures.

Figure 11 shows the fractal dimension calculation results of crack growth path of dry tuff specimen at 128.5 s^-1^ strain rate. Figure 12 shows the fractal dimension changes of crack growth paths in dry and saturated tuff samples under different impact pressures.

It can be seen from Fig. 12 that the fractal dimension of the specimen increases with the increase of the strain rate. When the strain rate is 102.8 s^−1^, 127.5 s^−1^ and 157.9 s^−1^, the mean fractal dimensions of the dried specimens are 1.25, 1.35 and 1.46, respectively. When the strain rate is 104.0 s^−1^, 131.2 s^−1^ and 157.2 s^−1^, the mean fractal dimensions of the saturated specimen are 1.36, 1.42 and 1.70, respectively. With the increase of strain rate, the extent of crack propagation and the fractal dimension of the specimen increase significantly. Under the same strain rate, the mean fractal dimension of the saturated specimen is higher than that of the dry specimen, and the mean fractal dimension of the saturated specimen under 0.3 MPa, 0.4 MPa and 0.5 MPa air pressure is 1.09, 1.05 and 1.16 times of that of the dry specimen, respectively. The crack propagation degree of the saturated specimen is much higher than that of the dry specimen. The increase of strain rate and water saturation increase the complexity of rock crack growth. The increase of strain rate makes the external input energy increase, the energy used for plastic deformation of the specimen increases, and the plastic deformation degree of the specimen also increases. Although the energy absorption effect of saturated rock mass is lower than that of dry specimens, due to the degradation of water, the deformation ability of the specimens to resist external loads is reduced, and the specimens are more prone to fracture and fracture under external impact loads, so the crack propagation degree of the specimens is significantly higher than that of dry specimens.

**Figure 13:**
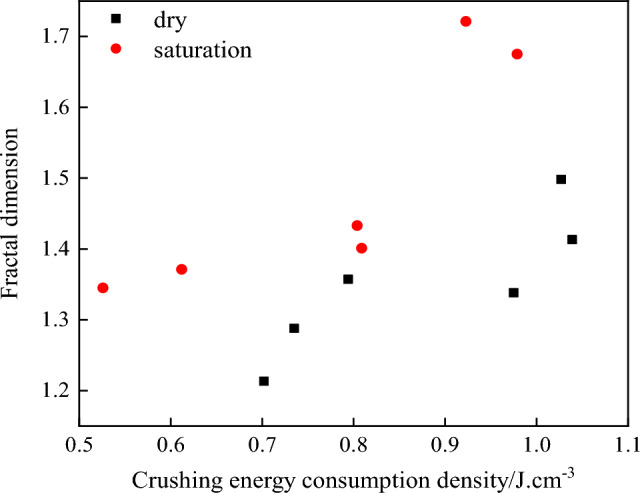
The relationship between the energy dissipation density and the fractal dimension.

In order to further analyze the relationship between the energy absorption effect of dry and saturated specimens and the extent of crack propagation, the relationship between the specific energy absorption value of specimens under different strain rates and the fractal dimension is treated, as shown in Fig. 13.

It can be seen from Fig. 13 that the crushing energy consumption density of the dry specimen is generally higher than that of the saturated specimen. Combined with Table 3, it can be seen that the mean energy dissipation density of water-saturated specimens under pressure of 0.3 MPa, 0.4 MPa and 0.5 MPa is 0.79, 0.91 and 0.92 times that of dry specimens, respectively, but the fractal dimension of water-saturated specimens is significantly higher than that of dry specimens. When the energy dissipation density reaches 0.98 J.cm^−3^, the fractal dimension of crack growth of dry and water-saturated specimens is 1.29 and 1.68, respectively, and the fractal dimension of water-saturated specimens is 1.30 times that of dry specimens. Water saturation reduces the energy absorption effect of rock mass and the ability to resist external loads, and increases the crack propagation degree. Under the same load, rock mass is more prone to failure. Therefore, for this mining project, the corresponding mining mode should be formulated in the flooded and saturated rock mass mining area to enhance the safety factor in the process of rock mass mining and prevent the excessive damage and deformation of rock mass in the process of blasting and tool excavation, which will lead to the occurrence of mining safety accidents.

## Conclusion

(i) Under static load, more than 90% of the energy of the specimen reached the peak stress is stored in the specimen as elastic energy, and the average energy of the water-saturated specimen is 67.55% of that of the dry specimen. The effect of water erosion and degradation makes the energy-storage and energy-absorption of the water-saturated specimen lower than that of the dry specimen.
(ii) The energy absorptivity and energy dissipation density of dry specimen under impact load are higher than those of saturated specimen. When the strain rate is about 102 s^-1^, the average energy consumption density of the saturated rock sample is 79.2% of that of the dry rock sample, and the average energy consumption density of the saturated rock sample is 92.1% of that of the dry rock sample at 155 s^-1^. Due to the existence of “Stefan” effect, the increase of energy dissipation density of water-saturated specimen at high strain rate is greater than that of dry specimen.
(iii) With the increase of strain rate and load time, the crack propagation degree increases. At the same strain rate, the number and width of cracks in water-saturated specimens are larger than that in dry specimens. Water-saturated specimens reduce the deformation resistance under external loads, and plastic deformation is more likely to occur under external loads.
(iv) The mean energy dissipation density of water-saturated specimens under 0.3 MPa, 0.4 MPa and 0.5 MPa is 0.79, 0.91 and 0.92 times of that of dry specimens, respectively, and the mean fractal dimension is 1.09, 1.05 and 1.16 times of that of dry specimens. The water-saturated rock mass significantly reduces the energy absorption effect, increases the fractal dimension of crack growth, and significantly reduces the resistance to external loads.

## Data Availability

The datasets used and/or analysed during the current study available from the corresponding author on reasonable request.
